# Genome analysis of *Spiroplasma citri* strains from different host plants and its leafhopper vectors

**DOI:** 10.1186/s12864-021-07637-8

**Published:** 2021-05-22

**Authors:** Rachel Rattner, Shree Prasad Thapa, Tyler Dang, Fatima Osman, Vijayanandraj Selvaraj, Yogita Maheshwari, Deborah Pagliaccia, Andres S. Espindola, Subhas Hajeri, Jianchi Chen, Gitta Coaker, Georgios Vidalakis, Raymond Yokomi

**Affiliations:** 1 Crop Diseases, Pests, and Genetics Research Unit, San Joaquin Valley Agricultural Sciences Center, USDA Agricultural Research Service, Parlier, CA 93648 USA; 2 Department of Plant Pathology, University of California, Davis, CA 95616 USA; 3 Department of Microbiology and Plant Pathology, University of California, Riverside, CA 92521 USA; 4 Department of Entomology & Plant Pathology and Institute of Biosecurity and Microbial Forensics, Oklahoma State University, Stillwater, OK 74078 USA; 5 Citrus Pest Detection Program, Central California Tristeza Eradication Agency, Tulare, CA 93274 USA

**Keywords:** Citrus stubborn disease, Beet leafhopper, Spiroplasma, Sequencing, Genome assembly, Prophage

## Abstract

**Background:**

*Spiroplasma citri* comprises a bacterial complex that cause diseases in citrus, horseradish, carrot, sesame, and also infects a wide array of ornamental and weed species. *S. citri* is transmitted in a persistent propagative manner by the beet leafhopper, *Neoaliturus tenellus* in North America and *Circulifer haematoceps* in the Mediterranean region. Leafhopper transmission and the pathogen’s wide host range serve as drivers of genetic diversity. This diversity was examined in silico by comparing the genome sequences of seven *S. citri* strains from the United States (BR12, CC-2, C5, C189, LB 319, BLH-13, and BLH-MB) collected from different hosts and times with other publicly available spiroplasmas.

**Results:**

Phylogenetic analysis using 16S rRNA sequences from 39 spiroplasmas obtained from NCBI database showed that *S. citri* strains, along with *S. kunkelii* and *S. phoeniceum*, two other plant pathogenic spiroplasmas, formed a monophyletic group. To refine genetic relationships among *S. citri* strains, phylogenetic analyses with 863 core orthologous sequences were performed. Strains that clustered together were: CC-2 and C5; C189 and R8-A2; BR12, BLH-MB, BLH-13 and LB 319. Strain GII3–3X remained in a separate branch. Sequence rearrangements were observed among *S. citri* strains, predominantly in the center of the chromosome. One to nine plasmids were identified in the seven *S. citri* strains analyzed in this study. Plasmids were most abundant in strains isolated from the beet leafhopper, followed by strains from carrot, Chinese cabbage, horseradish, and citrus, respectively. All these *S. citri* strains contained one plasmid with high similarity to plasmid pSci6 from *S. citri* strain GII3–3X which is known to confer insect transmissibility. Additionally, 17 to 25 prophage-like elements were identified in these genomes, which may promote rearrangements and contribute to repetitive regions.

**Conclusions:**

The genome of seven *S. citri* strains were found to contain a single circularized chromosome, ranging from 1.58 Mbp to 1.74 Mbp and 1597–2232 protein-coding genes. These strains possessed a plasmid similar to pSci6 from the GII3–3X strain associated with leafhopper transmission. Prophage sequences found in the *S. citri* genomes may contribute to the extension of its host range. These findings increase our understanding of *S. citri* genetic diversity.

**Supplementary Information:**

The online version contains supplementary material available at 10.1186/s12864-021-07637-8.

## Background

Spiroplasmas (helical mollicutes: Firmicutes: Mollicutes: Entomoplasmatales: Spiroplasmataceae) are wall-less, gram-positive bacteria with mobile helical cells. The bacteria are fastidious, culturable in cell-free media [1], and have a diverse host range [2]. Spiroplasmas are found in many arthropods including bees, flies, mosquitos, scorpion flies, beetles, and ticks [2] and have host relationships that range from commensal, mutualistic, and pathogenic [3]. Plant pathogenic spiroplasmas cause economic damage to crops and ornamentals. These pathogens include: *Spiroplasma citri,* causal agent of citrus stubborn disease (CSD) [4], brittle root of horseradish [5], and carrot purple leaf [6]; *S. kunkelli,* the causal agent of corn stunt [7]; and *S. phoeniceum*, isolated from periwinkle showing symptoms typical of mycoplasma-like organisms [8]. Plant pathogenic spiroplasmas are transmitted in a persistent propagative manner by leafhoppers. Vectors of *S. citri* are the beet leafhopper (BLH), *Neoaliturus* (syn. *Circulifer) tenellus* (Baker) [9] in North America and *Circulifer haematoceps* (Mulsant et Rey) in the Mediterranean region [10]. *S. kunkelli* is transmitted by *Dalbulus maidis* (DeLong) [7] and *S. phoeniceum* was experimentally transmitted by *Macrosteles fascifrons* (Stål) [8].

Characterization of spiroplasmas have been based on morphological and biological properties. However, because growth, metabolism, and DNA-DNA relatedness studies are time consuming, serological deformation tests and enzyme-linked immunosorbent assays have been used for identification of new spiroplasma groups in accordance to the International Subcommittee on the Taxonomy of Mycoplasmatales [2, 11]. Recently, long-read high-throughput sequencing technology and whole genome sequencing of bacteria have become cost-effective and offers a precise method to differentiate bacterial species and strains that have highly repetitive regions in its genome [12, 13].

The pathogen’s wide host range and vector transmission serve as bottlenecks and drivers of genetic diversity of *S. citri* populations. Although *S. citri* exist in free living form in insect hemolymph and appropriate culture media, the pathogen in the vector must enter and move through the salivary gland and exit into the salivary duct and be expelled by the vector during probing and/or feeding in plants where *S. citri* infects host phloem tissue and exists intracellularly and is phloem-limited. Therefore, the objective of this study is to examine the genomes of *S. citri* collected from diverse hosts from different locations and times; and analyze the relationship between the genotype and phenotype of *S. citri* from citrus and horseradish (perennial crops); carrot and Chinese cabbage (annual crops); and from the BLH vector. The analysis was performed on whole-genome sequences of seven newly sequenced strains of *S. citri* and compared amongst each other, other *S. citri* strains, and spiroplasmas from other hosts. New insights in the evolution and diversity of *S. citri* is presented herein.

## Results

### Genome assembly and annotation

Cultures of six strains of *Spiroplasma citri* were established and sequences reported previously (Table 1) [14, 15] and a new strain, C5, is reported here. Briefly, *S. citri* strains C189 and LB 319 were isolated from the woody crop, citrus. BR12, CC-2, and C5 strains were isolated from the seasonal crops such as horseradish, Chinese cabbage, and carrot, respectively. BLH-13 and BLH-MB strains were isolated from the BLH. The complete genomes of the six strains were acquired using the long-read technology, PacBio [14, 15] and C5 was obtained using Nanopore sequencing technology. Sequences from each strain were assembled into single chromosomal contigs. Contigs that did not associate with the chromosome were designated as putative plasmids (Table 2). The chromosome and plasmid status of each contig were further confirmed by BLASTn analyses against the GenBank database Release 236 (Supplementary Table S1). The circular chromosome for all seven strains ranged from 1,576,550 to 1,742,208 bp, with an average G + C content of 25.4%. Total genome size ranged from 1,611,714 to 1,832,173 bp in strains isolated from plants and 1,968,976 to 2,155,613 bp in strains isolated from the BLH. Annotation of each contig was performed by the NCBI Prokaryotic Genome Annotation Pipeline (PGAP), which predicted 32 tRNA genes, three rRNA genes and protein-coding genes which ranged between 1597 and 2232. Extrachromosomal DNAs, characterized as putative plasmids varied in all the strains viz., one or two plasmids from citrus, two plasmids from horseradish, three plasmids from Chinese cabbage, seven plasmids from carrot, and eight or nine plasmids from the BLH. Putative plasmid sizes ranged from 2047 bp to 135,023 bp (Supplementary Table S1). Seven of the 32 plasmids identified in these seven strains could not be circularized and further research is needed to determine if they are linear or products of sequencing error or culturing conditions.

**Table 1:** *Spiroplasma citri* strains analyzed in this study

Strain	Host	Location	Year of collection	Reference
C189	Citrus	Riverside, California	1960	[14]
LB 319	Citrus	Ducor, California	2007	[14]
BR12	Horseradish	Collinsville, Illinois	1984	[14]
CC-2	Chinese cabbage	Fresno, California	2016	[15]
C5	Carrot	Bakersfield, California	2005	This study
BLH-13	Beet leafhopper	Mettler, California	2010	[14]
BLH-MB	Beet leafhopper	Parlier, California	2011	[14]

**Table 2:** Genome assembly statistics for *Spiroplasma citri* strains analyzed in this study

***Spiroplasma citri*** strain	***S. citri*** C189	***S. citri*** LB 319	***S. citri*** BR12	***S. citri*** CC-2	***S. citri*** C5	***S. citri*** BLH-13	***S. citri*** BLH-MB
**Chromosome size (bp)**	1,577,041	1,734,522	1,731,112	1,709,192	1,618,536	1,576,550	1,742,208
**Combined size of plasmids (bp)**	34,663	95,488	101,061	82,444	126,436	392,426	413,405
**Chromosome + plasmid size (bp)**	1,611,704	1,830,010	1,832,173	1,791,636	1,795,359	1,968,976	2,155,613
**No. of plasmids**	1	2	2	3	7	8	9
**Chromosome GC content (%)**	25.6	25.4	25.4	25.6	25.6	25.4	25.4
**Total no. genes**	1946	2207	2200	2068	2064	2594	2411
**Total protein-coding genes**	1597	1853	1876	1716	1701	2232	2082
**Total rRNA genes**	3	3	3	3	3	3	3
**Total tRNA genes**	32	32	32	32	32	32	32
**GenBank Accessions**	CP047426.1, CP047427.1 [15]	CP046371.1 - CP046373.1 [15]	CP046368.1 - CP046370.1 [15]	CP042472.1 - CP042475.1 [14]	CP053304.1 - CP053311.1 (this study)	CP047428.1 - CP047436.1 [15]	CP047437.1 - CP047446.1 [15]

### Phylogenomics

**Fig. 1:**
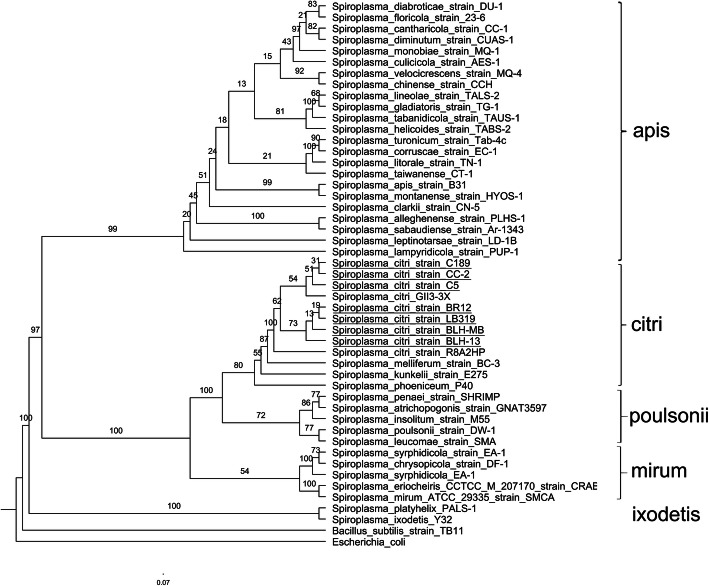
Maximum-likelihood 16S rRNA phylogenetic analysis of *Spiroplasma* species. A maximum-likelihood approach was used to generate the phylogeny with 1000 bootstrap replicates. Bootstrap values are indicated at each node. The resulting phylogeny was visualized using FigTree v. 1.4.3 [16]*. S. citri* strains analyzed in this report are underlined

**Fig. 2:**
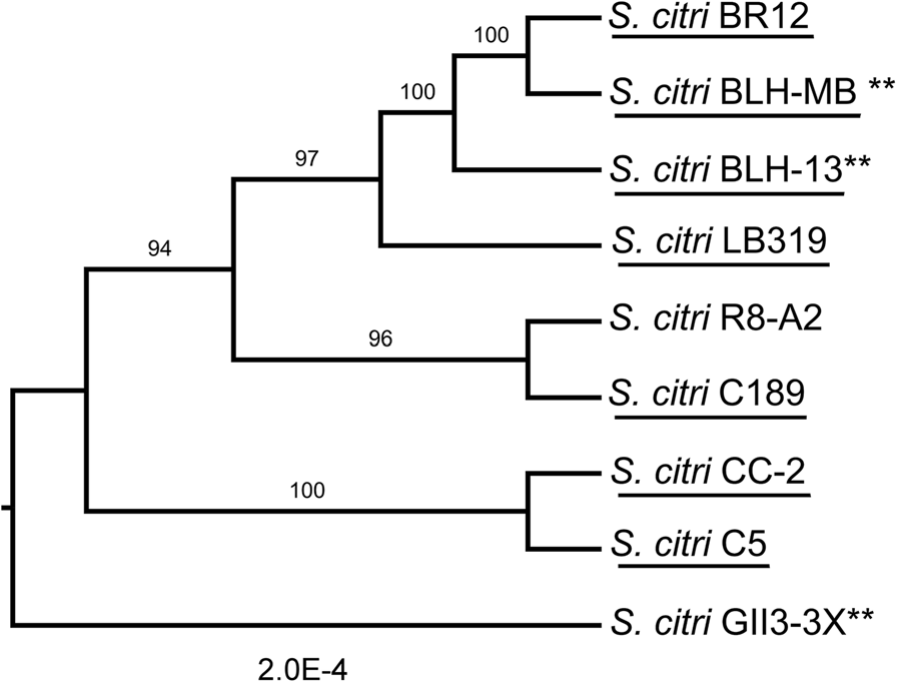
Phylogenetic analysis of *Spiroplasma citri* strains. Maximum-likelihood phylogeny of *Spiroplasma* based on core orthologous genes. In total, 863 orthologous genes were concatenated, and a maximum-likelihood approach was used to generate the phylogeny with 1000 bootstrap replicates. Bootstrap values are indicated at each node. The resulting phylogeny was visualized using FigTree v. 1.4.3 [16]. *S. citri* strains analyzed in this report are underlined. Strains isolated from beet leafhopper have been marked with asterisks (**)

Molecular phylogenetic inference of 39 spiroplasmas was performed using 16S rRNA genes in the NCBI database. Analysis of this gene sequence indicated that *S. citri* strains are closely related, but not identical. The phylogeny inferred from the 16S rRNA gene shows that *S. citri* strains formed a monophyletic group with plant pathogenic *S. kunkelii, S. phoeniceum,* and a honeybee pathogen, *S. melliferum* (Fig. 1, Supplementary Table S2). To facilitate a high-resolution comparison of *S. citri* strains, core genomes were analyzed for nine *S. citri* genomes available in NCBI. Using the orthoMCL pipeline, a total of 863 orthologous genes were identified as conserved among the *S. citri* strains. The 863 orthologous genes were concatenated, and a maximum-likelihood approach was employed to generate a *S. citri* phylogeny (Fig. 2). Phylogenetic analyses with the core orthologous sequences among the *S. citri* strains showed citrus strains C189 from southern California and R8-A2 from Morocco clustered together. CC-2, isolated from Chinese cabbage, and C5, isolated from carrot, clustered together. Strains LB 319, BLH-13, BLH-MB, and BR12 clustered together in a separate clade. There was clear separation of *S. citri* from *S. kunkelii* (Supplementary Fig. S1).

### Comparative genomics

**Fig. 3:**
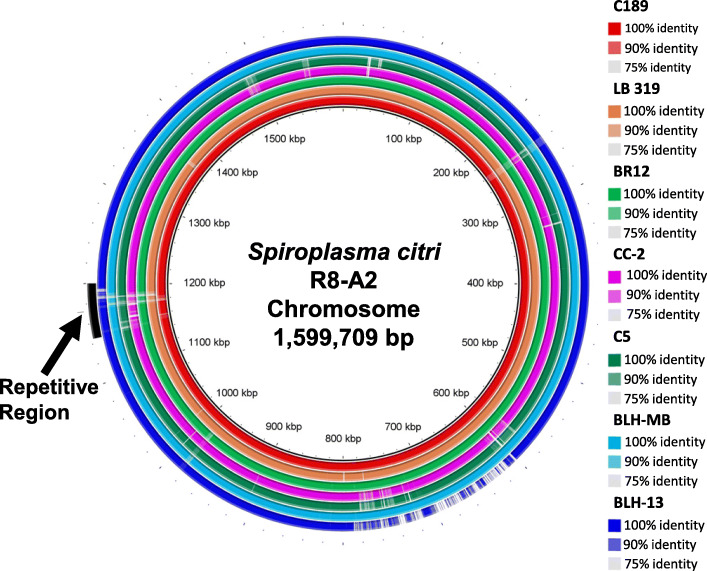
BLASTn results of *Spiroplasma citri* chromosomes. Whole genome comparison of eight *S. citri* strains visualized by BLAST Ring Image Generator (BRIG) [17]. *S. citri* strain R8-A2 was used as a reference. Each of the illustrated rings refers to one *S. citri* strain’s chromosome, according to the listed coloration. White regions represent dissimilarity from the reference genome. A highly repetitive region, marked in this image, was revealed during dot-matrix pairwise sequence comparisons (Supplementary Fig. S2). This area is marked based on the location of this region identified in the R8-A2 strain

The circular chromosome of the seven *S. citri* strains from the U.S. was compared via BLASTn to the R8-A2 strain from citrus in Morocco as the reference sequence. Visualization of these results was performed using the BLAST Ring Image Generator (BRIG). This genome level comparison among *S. citri* strains isolated from different sources showed a high level of homogeneity among each other and the reference genome, R8-A2 (Fig. 3). *S. citri* strains C189, LB 319, and BR-12, which were isolated from citrus and horseradish, appear most similar to R8-A2. A large region of dissimilarity near the middle of the chromosome is notable in the BLH-13 strain, ranging from ~ 600 kbp to ~ 800 kbp. These differences were not found in the BLH-MB strain, but some variability in this region can be seen in CC-2 and C5.

Dot-matrix pairwise sequence comparisons revealed a highly repetitive region at ~ 1200 kbp (Supplementary Fig. S2). The repetitive region, marked in Fig. 3, is an area of higher dissimilarity in the chromosome among the *S. citri* strains analyzed in this study.

**Fig. 4:**
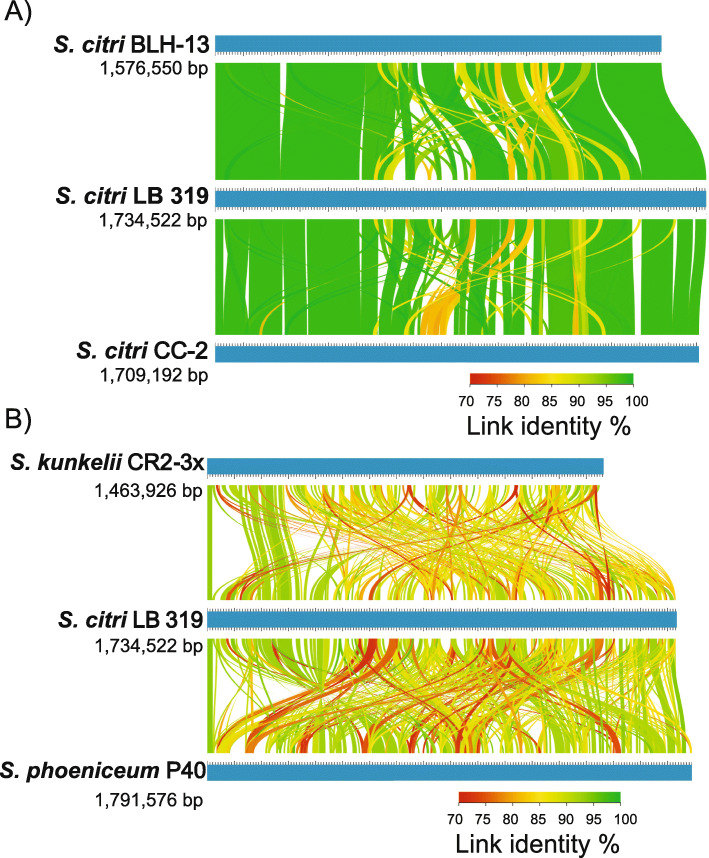
Genome-wide comparison of pathogenic *Spiroplasma* species. Linear chromosomal maps were built using AliTV v. 1.0 visualization software [18], based on whole-genome alignments with Lastz v. 1.0.4 aligner [19]. Both panels depict pairwise comparisons, expressed as percentage of nucleotide similarity, that connect different homologous genomic regions. Chromosomes are completely finished and pictured in blue. **a** *S. citri* strains BLH-13 isolated from the beet leafhopper (BLH), LB 319 isolated from citrus and CC-2 isolated from Chinese cabbage. **b** *S. citri* LB 319, *S. phoeniceum* P40 and *S. kunkelii* CR2-3X

Pairwise whole genome comparisons were performed with *S. citri* strains BLH-13, LB 319, and CC-2 which were selected to represent the biological diversity in this study. This comparison revealed high genome similarity, higher numbers of shared genes, and limited genome re-arrangements as observed in the center region (Fig. 4a). In contrast, genome comparison among different species of plant pathogenic spiroplasmas were examined using LB 319 from citrus, *S. phoeniceum* P40 from periwinkle, and *S. kunkelii* CR2-3X from corn. Here, *S. citri, S. kunkelii,* and *S. phoeniceum* showed significant differences in gene content, low level of genome similarity, and extensive genome rearrangements. *S. kunkelii* and *S. phoeniceum* also exhibited fewer regions of genome similarity and extensive genomic rearrangements (Fig. 4b).

**Fig. 5:**
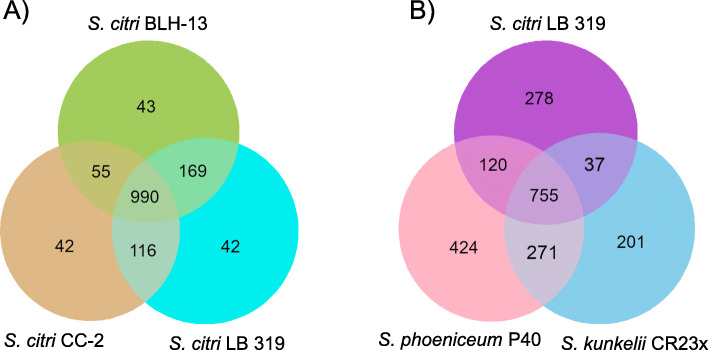
Numbers of shared and genome-specific homologous gene clusters. The Venn diagrams show the number of shared and genome-specific homologous gene clusters among the genomes compared. **a** Comparison among *Spiroplasma citri* BLH-13, CC-2, and LB 319. **b** Comparison among *S. citri* LB 319, *S. phoeniceum* P40, and *S. kunkelii* CR2-3X

Homologous genes were also identified among LB 319, BLH-13, and CC-2 (Fig. 5a). These *S. citri* strains shared 990 core homologous gene clusters, with 42 and 43 homologous gene clusters specific to each of these strains. Among different plant pathogenic *Spiroplasma* spp., LB 319, *S. phoeniceum* P40, and *S. kunkelii* CR2-3X, shared 755 core homologous gene clusters (Fig. 5b). There were 201 to 424 homologous gene clusters specific to *S. phoeniceum* P40 and *S. kunkelii* CR2-3x, respectively. LB 319 shared 120 homologous gene clusters with *S. phoeniceum,* while sharing only 37 homologous isolated from *S. kunkelii.* Additionally, *S. phoenicium* and *S. kunkelii* shared 271 homologous gene clusters that were absent from LB 319.

### Functional assignment of *S. citri* LB 319 protein-coding sequences

**Fig. 6:**
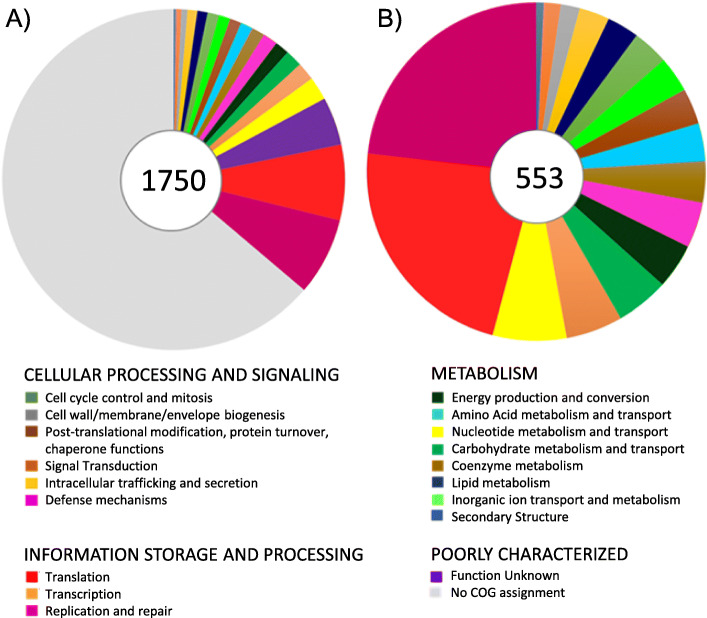
Functional classification of protein-coding genes in *S. citri* LB 319. The functional categorization of each protein-coding gene was classified according to the COG assignments. The pie graph indicates the COG distribution statistics. Each color represents a COG functional description. **a** All annotated protein-coding genes in the *S. citri* LB 319 genome. **b** Protein-coding genes that could be assigned functional category

Due to similarity of the chromosome of the seven *S. citri* strains studied, LB 319 was selected for further characterization. LB 319 had 1750 annotated protein coding sequences (CDS) and the functional classification of these protein coding sequences assigned only 553 CDS (32%) in different clusters of orthologous groups (COGs). The most abundant functional category was DNA replication, recombination and repair, followed by translation. These categories mainly consist of gDNA polymerases (*dnaE, dnaN, dnaX, holA, holB, polC*), nucleotide excision repair (*uvrA, uvrB, uvrC*), DNA topoisomerases (*gyrA, gyrB, parC, parE*), ribosomal proteins, and tRNA synthetases genes. Other important functional categories include translation (COG category K), nucleotide metabolism and transport (COG category F), and transcription (COG category O) (Fig. 6). Descriptive functional information of the genes is included in Supplemental Table S3. The low number of assigned COGs suggests that a large proportion of them may be fragments of unrecognized pseudogenes. Genes involved in mismatch repair like *mutS, mutI, mutH, exoI, exoX, recI* and genes involved in homologous recombination like *recA, recB, recC* etc. are missing or truncated.

### Plasmid variability between *S. citri* strains

**Fig. 7:**
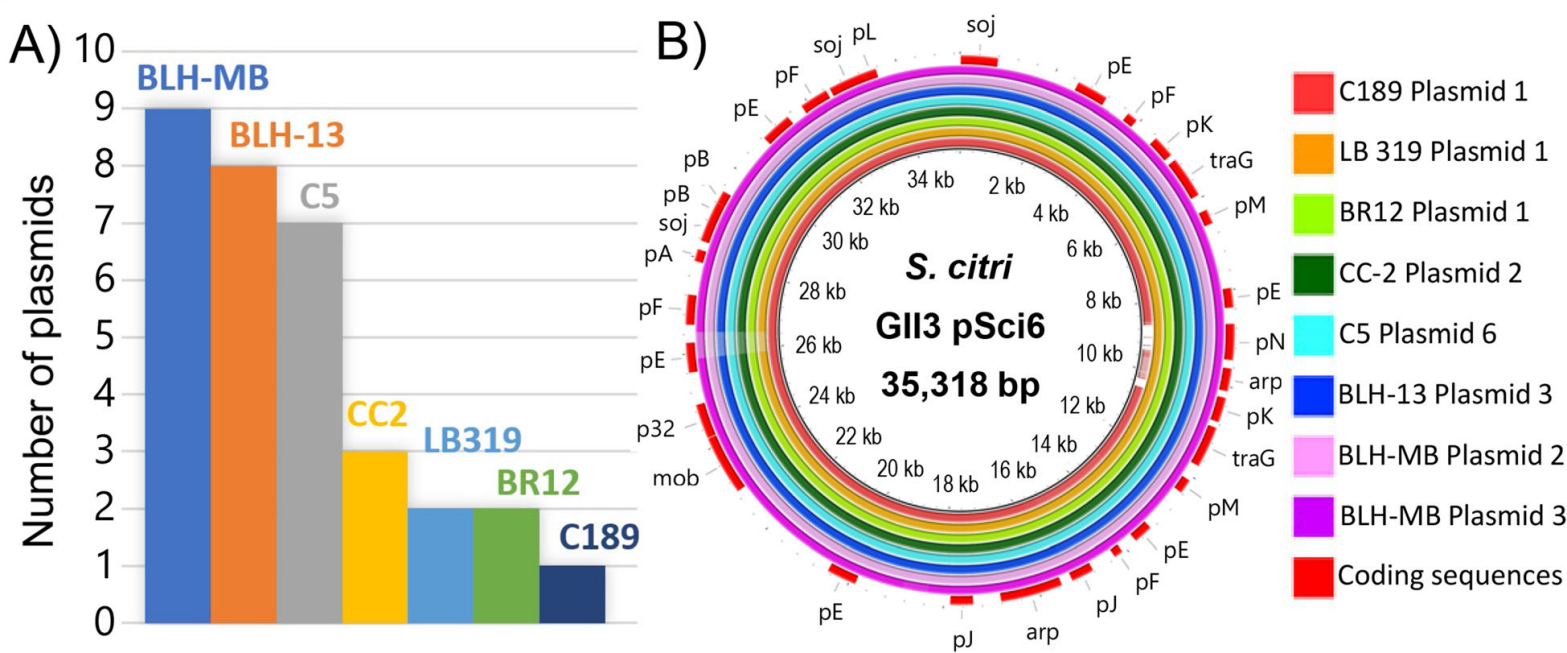
Analysis of *Spiroplasma citri* plasmids. **a** Number of plasmids from each of the *S. citri* strains analyzed in this study. Each bar represents one strain and is labeled with the host that strain was collected from. **b** BLASTn results of conserved *S. citri* plasmid. Whole genome comparison of conserved *S. citri* plasmid visualized by BLAST Ring Image Generator (BRIG) [17]. Plasmid pSci6, identified in *S. citri* strain GII3–3X [24], was used as a reference. Each of the illustrated rings refers to *S. citri* plasmids identified in the seven other strains analyzed in this study that were closely related to pSci6. Lighter regions represent dissimilarity from the reference plasmid. Outermost ring denotes coding sequences and is labeled with gene names. pA-pN represent hypothetical proteins, as named in GenBank accession AJ969074 [24]

Plasmids are genetic elements which may encode virulence factors and also play important roles in establishing host range [20–23]. Although a high level of similarity was found in the chromosome of *S. citri* strains, more diversity was found in the number of putative plasmids associated with these strains (Fig. 7a). For the sake of this study these putative plasmids are referred to as plasmids since the DNA in 25 of 32 plasmid-like contigs were circularized. Eight and nine plasmids were found in *S. citri* strains isolated from the BLH. Strains isolated from carrot and Chinese cabbage contained seven and three plasmids, respectively. *S. citri* strains isolated from citrus and horseradish possessed one to two plasmids. *S. citri* adhesion-related proteins (ScARPs), which are expected to be involved in *S. citri* adhesion to insect cells [25, 26], were predicted in several plasmids by NCBI’s Prokaryotic Genome Annotation Pipeline (PGAP). These ScARPs were present in one plasmid in BR12 and CC-2, two plasmids in C5 and BLH-MB, and three plasmids in BLH-13. No full-length ScARPs were predicted in C189 or LB 319 plasmids. *S. citri* strain C189, isolated from citrus in 1960, retained only one plasmid, pScp-C189–1. BLAST results revealed that this plasmid was highly similar to plasmid pSci6, identified in *S. citri* strain GII3–3X [24]. All strains analyzed in this study contained at least one plasmid with very high similarity to pSci6. (Fig. 7b). BLH-MB possessed two plasmids which resembled pSci6.

### Prophage prevalence in *S. citri* strains

The PHASTER (PHAge Search Tool – Enhanced Release) web server was used to identify and annotate putative prophage regions within the *S. citri* genomes. Seventeen to 25 prophage-like elements were identified in the *S. citri* genomes (Supplementary Fig. S3). Plasmid pScpLB319–2 in LB 319 and pScpC5–3 in C5 contained prophage sequences (Supplementary Fig. S3 B,E). Two plasmids in BLH-13 (pSciBLH13–1 and pSciBLH13–6) and three plasmids in BLH-MB (pSciBLHMB-1, pSciBLHMB-7, and pSciBLHMB-8) possessed prophage sequences (Supplementary Fig. S3 F,G). No prophage sequences were predicted in plasmids from C189, BR12, or CC-2. A large proportion of these prophage sequences were homologous to plectrovirus SpV1 [27] and SVTS2 [28] (Supplementary Table S4). While multiple plectrovirus SpV1 sequences were found to be distributed throughout the chromosome, a concentrated area of SVTS2 sequences was found at approximately 1.2 Mbp. This region of the chromosome was found to have highly repetitive sequences in all *S. citri* genomes in this study as shown by dot-matrix pairwise sequence comparisons (Fig. 3, Supplementary Fig. S2). Further characterization of the prophage region in the chromosome and plasmids will be examined in the future.

### Putative virulence components in *S. citri*

*S. citri* does not have specialized protein secretion apparatus, such as the type II and type III secretion systems. *S. citri* utilizes a sec-dependent protein export pathway. In *S. citri* LB 319, *secY* (GMI18_RS01645), *secA* (GMI18_RS01115), *ftsY* (GMI18_RS00765), *ffh* (GMI18_RS02240), *secE* (GMI18_RS01245), and *yidC* (GMI18_RS10485) are the genes involved in the sec-dependent pathway and are conserved across *S. citri* strains. Several sequences in plasmid pScp-C189–1 were homologous to genes associated with type IV secretion systems, including Mob-like transmembrane proteins, TraG proteins, and proteins containing TraM recognition domains and type IV secretion system DNA-binding domains. This plasmid is conserved across *S. citri* strains. Fructose operon genes are major pathogenicity determinants in *S. citri* [29, 30]. *fruR* (GMI18_RS08785), *fruA* (GMI18_RS08780) and *fruK* (GMI18_RS08775) are genes present in the fructose operon and were present in all the *S. citri* strains analyzed in this study. *fruR* codes for bacterial transcriptional regulators of carbohydrate catabolic operons. *fruA* codes for the permease of the phosphoenolpyruvate:fructose phosphotransferase system. This fructose permease allows uptake and concomitant phosphorylation of fructose into fructose-1-phosphate. *fruK* codes for a 1-phosphofructokinase, which uses ATP to phosphorylate fructose-1-phosphate into fructose-1,6-bisphosphate [30].

## Discussion

Prior to advances in long-read sequencing, the best assembly of *S. citri* was strain GII3–3X, which contained 39 chromosomal contigs [24]. The first sequence of *S. citri* containing a single chromosomal contig was reported in 2017 for Moroccan *S. citri* strain R8-A2^T^ [31]. Complete genomes, with single chromosomal contigs, of six more strains of *S. citri* isolated in the United States were reported in 2020 [14, 15] and a seventh strain, C5, isolated from carrot, is reported in this study. Chromosome sizes of these genomes were similar in size to strain R8-A2^T^ (~ 1.6 Mbp), although most of the U.S. strains were slightly larger in size. Additionally, the number of predicted protein coding regions was higher in U.S. strains compared to the R8-A2^T^ genome. One set of rRNA genes and 32 tRNA genes were predicted in U.S. strains, which is consistent with the R8-A2 ^T^ strain [31].

Phylogenetic analyses between *S. citri* strains showed citrus strains C189 from southern California and R8-A2 from Morocco clustered together. C189 was originally obtained in 1957 from Washington Navel in Riverside, California by graft transmission to sweet orange seedling [4] and has been continuously maintained in the greenhouse. R8-A2 was originally obtained from Washington Navel in Morocco by graft transmission to sour orange [32]. Therefore, both strains were isolated and cultured from greenhouse citrus hosts many years after initially acquired and sequenced. Strains LB 319, BLH-13, BLH-MB, and BR12 clustered together. In these cases, *S. citri* was isolated and cultured directly from these field sources and sequenced. CC-2 and C5, which originated from annual hosts with similar row crop ecologies, clustered together in a separate clade. Moreover, the analysis of core orthologous genes suggested that strains from citrus were very closely related and BLH strains were more closely related to citrus strains than those from Chinese cabbage or carrot. *S. citri* strain GII3–3X, isolated from *C. haematopceps*, did not group with strains isolated from BLHs from California, but this may be due to the incomplete genome sequence of GII3–3X.

A high level of homology and synteny was observed between the *S. citri* strains in this study, with some dissimilar regions and genomic rearrangements appearing in the center region of the chromosome. However, comparison with the most closely related species, *S. phoeniceum* and *S. kunkelii,* showed that the chromosomal organization is largely rearranged and exhibits much lower levels of sequence similarity. *S. phoeniceum* is associated with lethal yellows in periwinkle [33], while *S. kunkelii* causes corn stunt disease of *Zea mays* L. [34]. Large rearrangements were also observed when comparing *S. citri* to *S. melliferum*, a honeybee pathogen [35]. When comparing gene content, *S. citri* strains shared approximately 80% of the homologous gene clusters observed, with about 3% of gene clusters being unique to each strain. However, *S. citri* strain LB 319 shared approximately 64% of gene clusters with *S. phoeniceum* and *S. kunkelii,* while about 23% of gene clusters were unique to *S. citri*. These differences may be caused by differential gene loss, phage-mediated horizontal gene acquisition, and by ecological and biological diversification [35, 36].

Moreover, some of this variation may be due to variation in prophage sequences [37], which are viral or phage genomic DNA sequences integrated into a bacterial genome. *S. citri* is highly susceptible to viral invasion, due to its lack of a cell wall [38]. Between seventeen and twenty-five areas of the genome were predicted to contain prophage insertions in the *S. citri* genomes studied which contributed to the variations in chromosome size. Most of these sequences observed in *S. citri* are homologous to SpV1, a plectrovirus, and were dispersed throughout the genome [37]. PHASTER analysis demonstrated that SpV1 was prevalent throughout the chromosome of all seven *S. citri* strains, but the number and positions were inconsistent (Supplemental Fig. S3). SpV1 viral sequences have been associated with major variations of the *S. citri* genome [39]. These viral sequences, integrated into the *Spiroplasma* chromosome, can have a large effect on genome stability. A model for the evolution of the *Spiroplasma* genome has been linked to viral invasion, which could account for intraspecific genome size variation, low conservation in chromosomal organization, and a gain of lineage-specific genes [36]. The rearrangements and genome instability are apparent in pairwise comparisons of *S. citri* strains, and even more so when compared to their closest relatives, *S. phoeniceum* and *S. kunkelii*. Viral invasions likely promoted these rearrangements in plant pathogenic bacteria and could attribute to their adaptation to different hosts. In contrast to SpV1, multiple copies of phage sequences homologous to SVTS2 were present in one specific, repetitive region in all the *S. citri* strains studied (Supplementary Fig. S3). Integration of SVTS2 viral sequences into the chromosome of *S. citri* has been associated with the resistance of *S. citri* to spiroplasma virus SVTS2 [40]. This may be why SVTS2 sequences are not prevalent throughout the genome and repeating elements containing SVTS2 sequences are conserved across strains.

*S. citri* does not have specialized protein secretion apparatus like the Type II and Type III secretion systems, but instead utilizes the sec-dependent protein export pathways S. citri does not have T4SS. Components of the T4SS are present and highly conserved in *S. citri* strains analyzed in this study. However, their importance in virulence has yet to be verified. T4SSs are related to bacterial conjugation systems and secrete substrates into a wide range of target cells, including other bacteria and eukaryotic cells. T4SSs are known to play a role in pathogenesis in a wide range of bacteria by genetic exchange and delivery of effector molecules to target cells [41].

Fructose operon genes are major virulence components in *S. citri*, which utilizes fructose for pathogenicity and growth in plants [29]. The fructose operon consists of three genes: *fruR*, *fruA*, and *fruK*. *S. citri* mutants of *fruR*, which likely encodes for the putative regulator protein of the fructose operon, abolished expression of all three genes of the operon. This mutant exhibited severely impaired pathogenicity, which could be restored when the mutant was complemented with functional fructose operon genes [30]. This work proposed an explanation of the role of fructose utilization in the pathogenicity of *S. citri*, suggesting that companion cells in the plant host compete for fructose. *S. citri* uses fructose as a carbon and energy source, resulting in a reduced fructose concentration in the plant companion cells, modifying the distribution of photoassimilates, leading to disease symptoms [30].

Every *S. citri* strain studied had a unique pattern of extrachromosomal DNA and the presence or absence, position (free or integrated), and number of these sequences is a significant source of variation among strains [42]. After several years of maintenance in plants, *S. citri* strain BR3-G showed chromosomal rearrangements compared to strain BR3-T, which was transmitted from plant to plant by the BLH [39]. Strain BR3-G was found to be non-transmissible by the BLH which was correlated to a large deletion of a SpV1-related transposase gene [39]. Prolonged cultivation of bacteria has been reported to cause free plasmid DNA to be integrated into the chromosome through recombination events [43–45]. Strains isolated from BLH examined in this study contained two or three plasmids with predicted prophage sequences. The strains isolated from plants exhibited one or no plasmids with predicted prophage sequences. The plasmids that contain viral sequences were homologous to those in the chromosome of *S. citri* strain R8-A2. It is plausible that after transmission by the BLH to plants, plasmids containing viral sequences could be incorporated by recombination with chromosomal plectroviral sequences. This would result in fewer plasmids in perennial plants compared to annual plants, which was observed in this study.

Plasmids of phytoplasmas and spiroplasmas are known to be involved in insect transmissibility [46–49]. All the newly sequenced *S. citri* strains contained at least one plasmid with high homology to plasmid pSci6, identified in *S. citri* strain GII3–3X [24]. pSci6 plasmid encodes P32 protein, associated with insect transmissibility, and this plasmid confers insect transmissibility into non-transmissible strains of *S. citri* [50]. P32 has been suggested to interact with surface membrane proteins and may be necessary, but not sufficient for spiroplasma adhesion and invasion of insect cells [51]. In strain GII-3, eight proteins belonging to the ScARP protein family, which are expected to be involved in *S. citri* adhesion to insect cells, were encoded by five plasmids [24–26]. In previous studies, a loss of the high-molecular-mass plasmids carrying ScARP genes was correlated with a non-transmissible phenotype [51]. ScARP genes were found to be present in plasmids isolated from perennial crops and the leafhopper vector in this study. These plasmids are highly similar to pSci2 and pSci5 from *S. citri* strain GII3–3X and pBJS-O from *S. citri* strain BR3–3X [24, 52] (Supplementary Table S1). Interestingly, no full-length ScARPs were predicted in C189 or LB 319 plasmids. The lack of additional plasmids containing ScARP genes, seen in strains obtained from perennial crops, suggest these strains could have lost their ability to be transmitted by the BLH.

Several other genes have been assigned putative functions in plasmid pSci6. This included *soj-parA*, which is involved in DNA partitioning, and *traG* and *mob*, which are associated with DNA transfer and are suggested to be involved in a Type IV secretory pathway [24]. A coding region was also identified in plasmid pScp-C189–1, which has homology to a plasmid replication-relaxation (relaxase) family protein. Plasmids in *S. citri* have been suggested to be horizontally transferred between cells by conjugation [24]. Several important genes are at the core of plasmid conjugation, including Type IV coupling proteins and relaxases [53]. The *traG* and *mob* genes found in pSci6 correspond to VirB4/D4 components of the type IV secretion pathway, which allows for the translocation of DNA through cytoplasmic membranes [54]. Walled bacteria require many components in their conjugation system; however, these components may not be necessary in *S. citri*, a wall-less bacterium. In addition to *traG* and *mob*, Saillard et al. suggested that pSci6 should contain a relaxase, but this family of proteins was not reported [24]. The replication-relaxation (relaxase) family protein is essential for plasmid replication and plasmid DNA relaxation, part of conjugative DNA transfer in bacteria [55–57]. A BLAST search of the coding sequences in pScp-C189–1 revealed homology to a replication-relaxation family protein. This further supports previous studies that reported genetic exchanges by a conjugation-like process in *S. citri* [58]. The genes identified in this plasmid encode for virulence-associated proteins involved in adhesion and conjugal DNA transfer. The occurrence of this persistent, conserved plasmid suggests it plays an important role in this pathogen.

## Conclusion

Six *Spiroplasma citri* genomes were published recently, but were not fully analyzed [14, 15]. Those sequences, along with C5, a strain de novo assembled in this study, greatly expanded the availability of *S. citri* genomes and allowed performance of extensive in silico comparative genomic studies that provide insights into this organism’s genetic diversity and evolution. An extremely high level of homogeneity was observed in the chromosomal contigs across *S. citri* strains. Variation in plasmid number may play an important role in insect transmission and virulence. Moreover, differences in genome size and stability appear to result from variations in number and site of plectroviral sequences inserted into the genome. These features likely contribute to *S. citri* adaptation to different hosts and transmissibility by leafhopper vectors. Further studies will be necessary to validate the roles of plasmids and viral insertion sequences in *S. citri* strains isolated from various hosts.

## Methods

### Strain isolation and DNA preparation

Cultures of *S. citri* strains CC-2, C189, BR-12, LB 319, BLH-13, and BLH-MB were isolated, and DNA extracted in a previous study [14, 15]. *S. citri* strain C5 was collected in 2005 from carrot growing in NW Bakersfield, California. Briefly, *S. citri* was isolated and grown in LD8 medium [59]. Later the cultures were triple cloned and stored at − 80 °C until further use. Cultures were re-established and total genomic DNA was extracted by CTAB [60] or by DNeasy Blood and Tissue Extraction kit (Qiagen). Collection details of *S. citri* in this study are listed in Table 1.

### Whole-genome shotgun sequencing

*S. citri* strain C5 was sequenced on the Oxford Nanopore (Oxford, United Kingdom) MinION platform [61]. The library was prepared with Oxford Nanopore (Oxford, United Kingdom) Rapid Barcoding Kit-SQK-RBK004 according to the manufacturer’s specifications. Data was collected using MinION Release 19.10.1. Bases were called using Guppy v.3.4.5 and the adapter screening and quality filtering of raw sequencing data were performed using Fastp v 0.20.0 [62]. Remaining *S. citri* strains’ genomes were sequenced previously on PacBio (Menlo Park, CA, USA) RS II platform [14, 15].

### De novo sequencing assembly

Sequences from *S. citri* strains CC-2, C189, BR-12, LB 319, BLH-13, and BLH-MB were reported previously [14, 15]. Briefly, raw reads were filtered, subreads were established by PacBio, and assembled into contigs using Canu 1.8 [63]. For *S. citri* strain C5, contigs were assembled using Canu 1.8. An additional polishing step was performed by medaka v 1.0.3 (Oxford Nanopore Technologies) and frame-shift-corrected by DIAMOND v 0.9.28 [64] and MEGAN v 6.18.4 [65], following the pipeline described by Arumugam et al. [66]. Approximately 500 bp segments from each end of a contig were used for BLASTn search to check the contig singularity. Appropriate reads connecting both ends were used for enclosure. The chromosome and plasmid status of each contig were further confirmed by BLASTn analyses against the GenBank database. The genome sequence data was deposited in the NCBI database (accession numbers shown in Table 2). Annotation of each contig was performed by the NCBI Prokaryotic Genome Annotation Pipeline (PGAP) [67].

### Phylogenetic analyses

The 16S rRNA sequences of thirty-nine *Spiroplasma* species were obtained from the NCBI database. Sequence alignments were carried out with the PRANK alignment tool [68]. Maximum-likelihood approach was used to reconstruct the phylogenetic tree using RAxML software [69]. Bootstrapping was performed with 1000 replicates. The resulting phylogeny was visualized with FigTree [16].

Orthologous genes of *S. citri* isolates were predicted using the OrthoMCL v. 2.0 pipeline [70]. All-versus-all BLASTN (*E* value < 10^− 5^, alignment coverage > 50%) comparison of all gene sequences for each species was performed and orthologous genes were clustered by OrthoMCL v. 2.0. Multiple sequence alignment was done with PRANK v. 170,427 [71]. The sequence alignments were concatenated by FASconCAT v. 1.1, yielding a gene super-matrix [72]. Maximum-likelihood approach was used to reconstruct the phylogenetic tree using RAxML v. 8.2 software with 1000 bootstrap replicates [69]. The resulting phylogeny was visualized using FigTree v. 1.4.3 [16].

### Bioinformatics analysis

Large genome comparison of eight *S. citri* sequences was computed and visualized with the use of BLAST Ring Image Generator (BRIG) v 0.95 [17]. Pairwise genome alignment was achieved by the Lastz v. 1.04 program [19]. The results were visualized using AliTV v. 1.0 [18]. Shared and genome-specific genes were identified between the *S. citri* strains isolated from different sources and among *S. citri* LB 319, *S. kunkelii* CR2-3X, and *S. phoeniceum* P40. The sequence similarity search step in the OrthoMCL analysis was conducted at the nucleotide level [70]. Functional annotation of COG was done using eggNOG-mapper [73]. Prophage sequences were predicted using PHASTER online server [74].

## Supplementary Information

**Additional file 1: Supplementary Table S1.** BLASTn similarity search of *Spiroplasma citri* putative plasmid (contigs) against available *Spiroplasma* sequences deposited in NCBI database.

**Additional file 2: Supplementary Table S2**. GenBank accession numbers for the 16S rRNA *Spiroplasma* sequences used for phylogenetic analyses in Fig. 1.

**Additional file 3: Supplementary Table S3**. COG functional categories of *Spiroplasma citri* LB319

**Additional file 4: Supplementary Table S4**. Summary of PHAge Search Tool Enhanced Release (PHASTER) analysis of *Spiroplasma citri* strains

**Additional file 5: Supplementary Fig. S1**. Phylogenetic analysis of *Spiroplasma* strains. Maximum-likelihood phylogeny of *Spiroplasma* based on core orthologous genes. In total, 601 orthologous genes were concatenated, and a maximum-likelihood approach was used to generate the phylogeny with 1000 bootstrap replicates. Bootstrap values are indicated at each node. The resulting phylogeny was visualized using FigTree v.1.4.3 [70]. *S. citri* strains analyzed in this report are underlined.

**Additional file 6: Supplementary Fig. S2**. Dot-Matrix representation of a BLASTn comparison of complete *Spiroplasma citri* genome sequences. The chromosome of each newly assembled strain (X-axis) was compared to the reference sequence, strain R8-A2 (Y-axis). A) C189; B) LB 319; C) BR12; D) CC-2; E) C5; F) BLH-13; G) BLH-MB.

**Additional file 7: Supplementary Fig. S3**. Predicted prophage sequences in *Spiroplasma citri* genomes. Prophage annotation was performed by PHASTER (PHAge Search Tool – Enhanced Release). The program predicts the completeness of the predicted prophages, based on the proportion of phage genes in the identified region. Green bars are scored as intact (score > 90); blue bars are questionable (score 70–90); red bars are incomplete (score < 70). Strains with plasmids predicted to contain phage genes are included (Fig. S3B, E, F, and G). Approximate location of repetitive region identified by dot-matrix pairwise sequence comparisons (Supplementary Fig. S2) is highlighted in yellow.

## Data Availability

The data described in this study can be freely and openly accessed from the NCBI database. All sequence data has been deposited in GenBank under the accession numbers CP042472-CP042475, CP0426368-CP046373, CP047426-CP047446, and CP053304-CP053311. The versions described in this paper are the first versions. PacBio sequencing reads for BioProject PRJNA558054 have been deposited in the NCBI Sequence Read Archive (SRA) under accession number SRR9903453 for CC-2. PacBio sequencing reads for BioProject PRJNA591027 have been deposited in the NCBI SRA under accession numbers SRR10843927 for C189; SRR10507068 for LB 319; SRR10507067 for BR12; SRR10507065 for BLH-13; and SRR10507066 for BLH-MB. Nanopore sequencing reads for BioProject PRJNA625113 have been deposited in the NCBI SRA under accession number SRR11536473 for C5. *S. citri* cultures for BLH-13, BLH-MB, BR12, LB 319, and CC-2 have been deposited in ATCC no. SD-7532-7535 and SD-7278, respectively. However, ATCC Biorepository Service Agreement 2019-BRS-00049 maintains confidentiality of information regarding these cultures. C189 and C5 cultures are available from the corresponding author.

## Abbreviations

PGAP: Prokaryotic Genome Annotation Pipeline
CSD: citrus stubborn disease
CC: Chinese cabbage
LB: Liberty Bell
BR: brittle root
BLH: beet leafhopper
BRIG: BLAST Ring Image Generator
COG: cluster of orthologs groups
ScARPs: *S. citri* adhesion-related proteins
PHASTER: PHAge Search Tool – Enhanced Release
