# Supplementary figures and images for: Genome analysis of Spiroplasma citri strains from different host plants and its leafhopper vectors
Source: BMC Genomics. 2021 May 22;22:373. doi: 10.1186/s12864-021-07637-8 (PMC8140453; doi:10.1186/s12864-021-07637-8)

Supplementary Figure S1

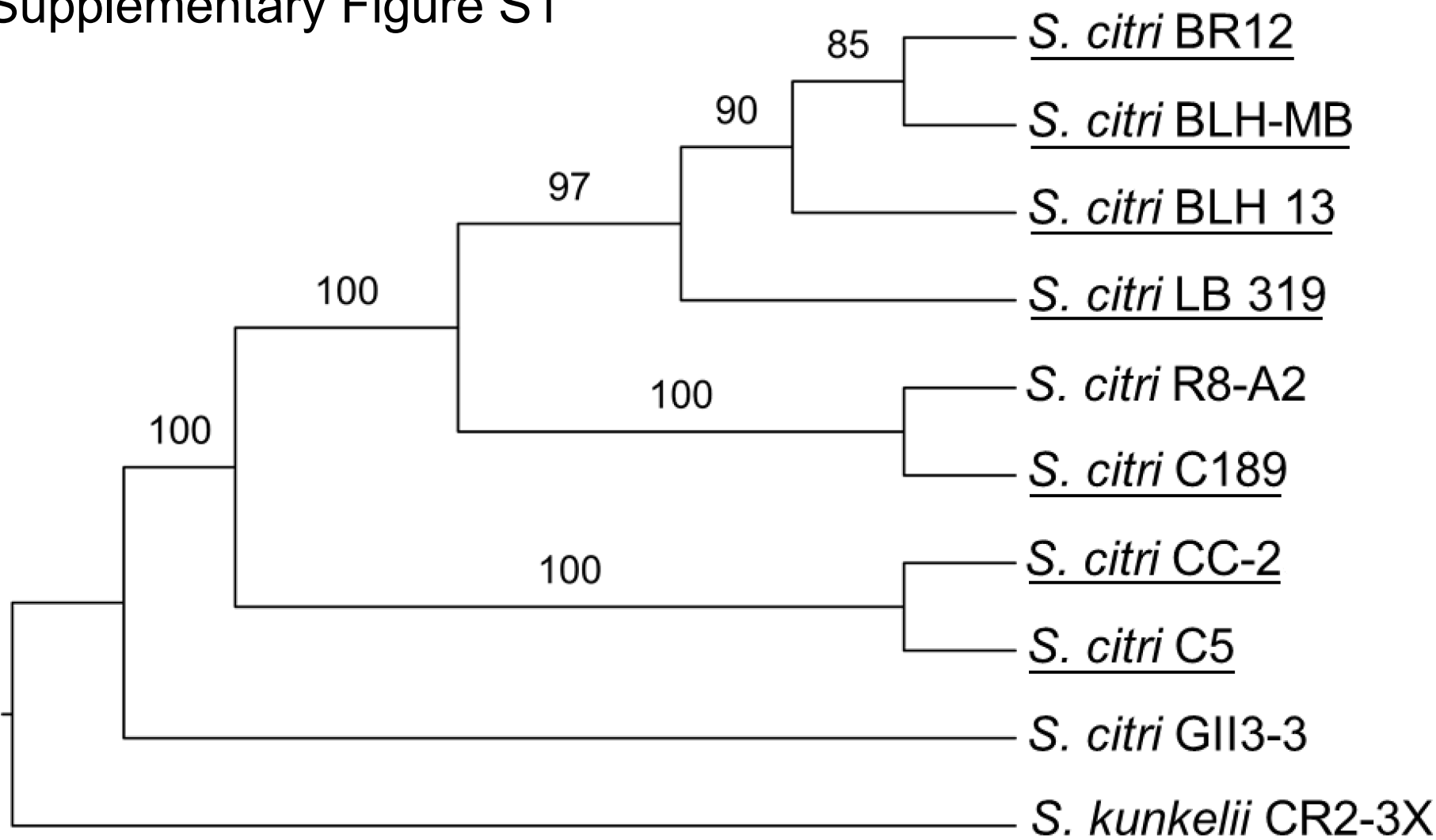

0.008

Supplement: Supplementary file 5 — Additional file 5: Supplementary Fig. S1. Phylogenetic analysis of Spiroplasma strains. Maximum-likelihood phylogeny of Spiroplasma based on core orthologous genes. In total, 601 orthologous genes were concatenated, and a maximum-likelihood approach was used to generate the phylogeny with 1000 bootstrap replicates. Bootstrap values are indicated at each node. The resulting phylogeny was visualized using FigTree v.1.4.3 [70]. S. citri strains analyzed in this report are underlined. [file 12864_2021_7637_MOESM5_ESM.pdf]

# Supplementary Figure S2

A

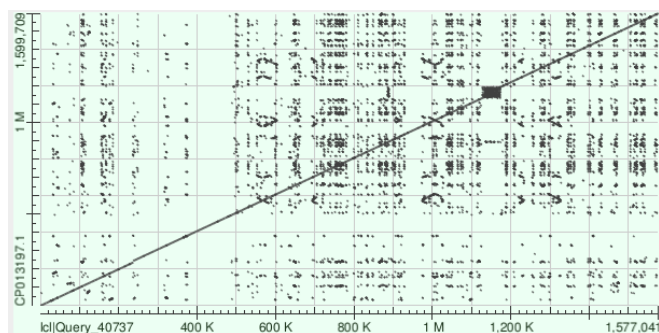

B

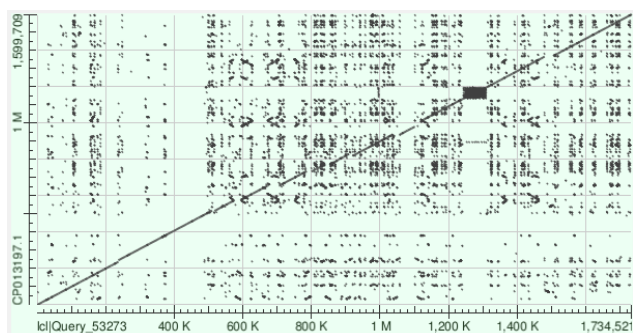

C

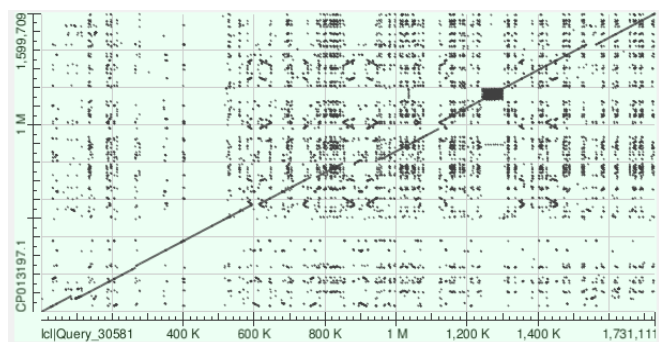

D

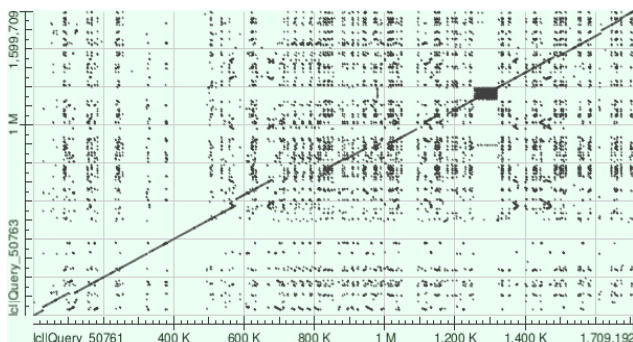

E

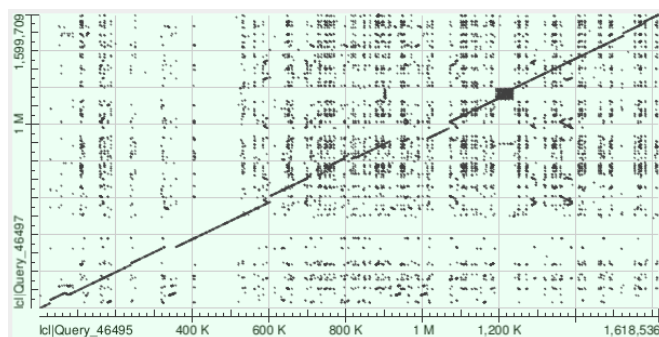

F

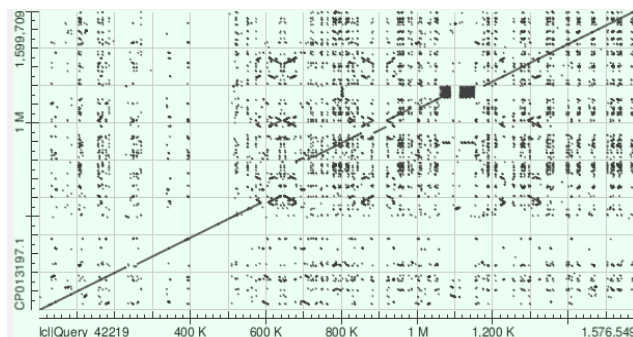

G

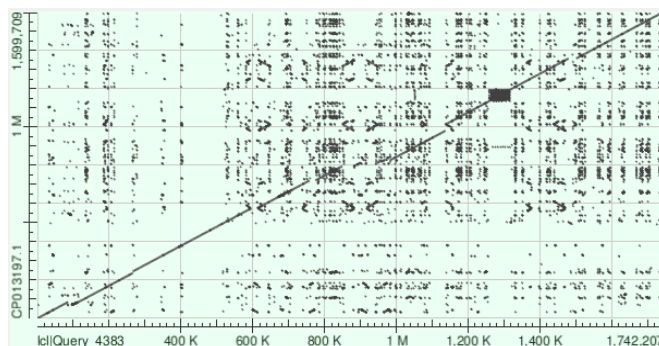

Supplement: Supplementary file 6 — Additional file 6: Supplementary Fig. S2. Dot-Matrix representation of a BLASTn comparison of complete Spiroplasma citri genome sequences. The chromosome of each newly assembled strain (X-axis) was compared to the reference sequence, strain R8-A2 (Y-axis). A) C189; B) LB 319; C) BR12; D) CC-2; E) C5; F) BLH-13; G) BLH-MB. [file 12864_2021_7637_MOESM6_ESM.pdf]

# Supplementary Figure S3

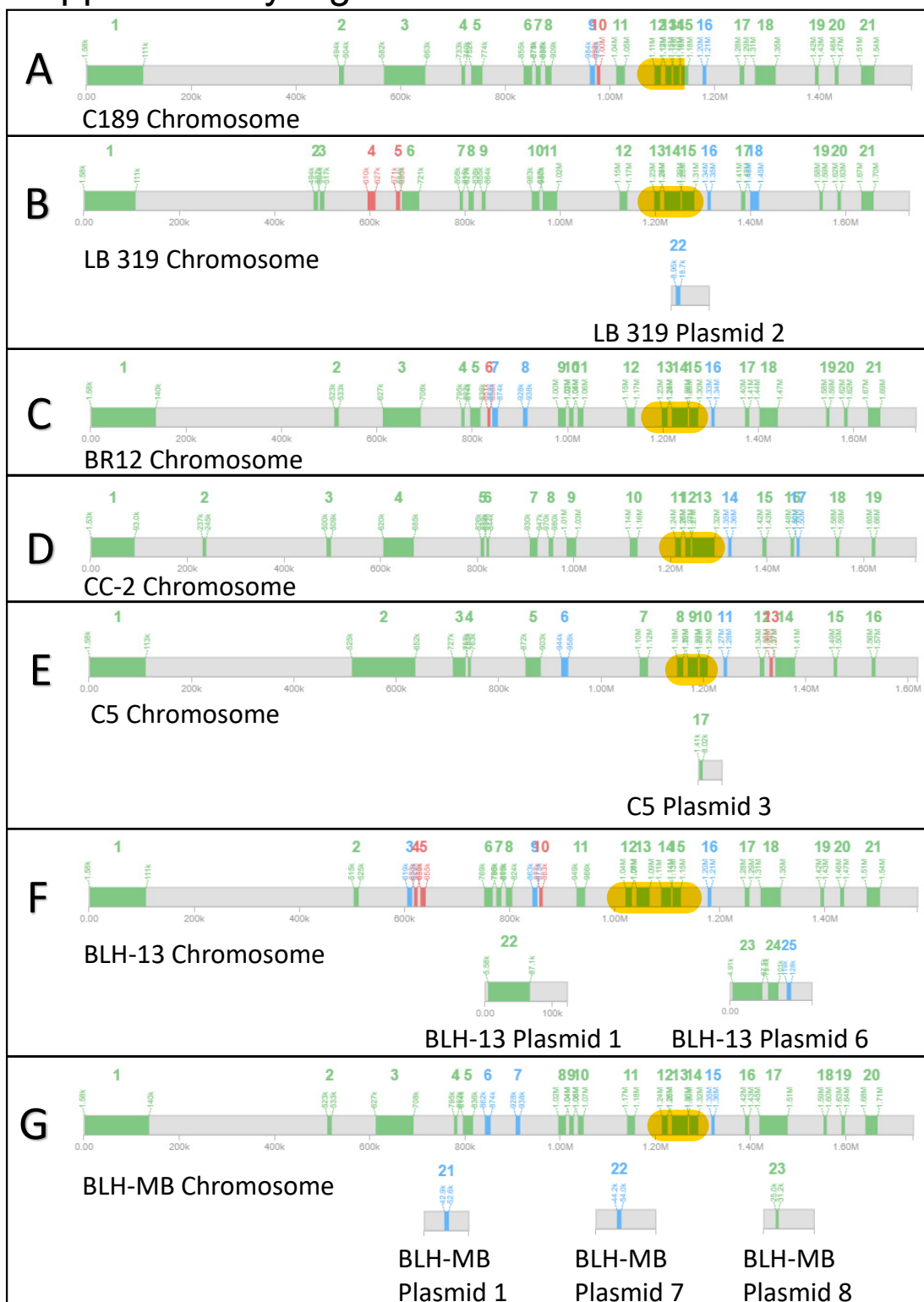

Supplement: Supplementary file 7 — Additional file 7: Supplementary Fig. S3. Predicted prophage sequences in Spiroplasma citri genomes. Prophage annotation was performed by PHASTER (PHAge Search Tool – Enhanced Release). The program predicts the completeness of the predicted prophages, based on the proportion of phage genes in the identified region. Green bars are scored as intact (score > 90); blue bars are questionable (score 70–90); red bars are incomplete (score < 70). Strains with plasmids predicted to contain phage genes are included (Fig. S3B, E, F, and G). Approximate location of repetitive region identified by dot-matrix pairwise sequence comparisons (Supplementary Fig. S2) is highlighted in yellow. [file 12864_2021_7637_MOESM7_ESM.pdf]
